# COVID-19 and Mental Health of Minority Arab Higher-Education Students in Israel: Social, Economic, and Academic Factors

**DOI:** 10.3390/ijerph192013466

**Published:** 2022-10-18

**Authors:** Samira Alfayumi-Zeadna, Lena Gnaim-Abu Touma, Maya Weinreich, Norm O’Rourke

**Affiliations:** 1 Nursing Department, School of Health Sciences, Ashkelon Academic College, Ashkelon 78211, Israel; 2 MAP Centre for Urban Health Solutions, Li Ka Shing Knowledge Institute, Michael’s Hospital, Unity Health Toronto, Toronto, ON M5B 1W8, Canada; 3 Department of Education, Al-Qasemi Academic College of Education, Baqa-El-Gharbia 30100, Israel; 4 Medical School for International Health, Faculty of Health Sciences, Ben-Gurion University of the Negev, Beer Sheva 84105, Israel; 5 School of Public Health and Multidisciplinary Center for Research on Aging, Ben-Gurion University of the Negev, Beer Sheva 84105, Israel; 6 Department of Psychology, Ben-Gurion University of the Negev, Beer Sheva 84105, Israel

**Keywords:** COVID-19, mental health, depression, anxiety, stress, higher-education students, ethnic minorities, Arab students

## Abstract

The mental health and well-being of higher-education students is a topic of growing interest. COVID-19 impacted higher education in many ways and the challenges were especially pronounced for minority students. This study examines the impact of COVID-19 on the mental health of Arab minority students in Israel in relation to social, academic, and financial factors. We recruited 420 Arab higher-education students enrolled in academic colleges or universities in Israel who completed a battery of online questionnaires. Mental health status was measured by the Depression, Anxiety, and Stress Scale 21 (DASS-21). Moderate to severe symptoms of depression, anxiety, and stress were reported by 49.3%, 45.2%, and 54% of Arab students, respectively. Analyses indicate that low quality of online learning, academic difficulties, and negative economic effects of COVID-19 predicted stress, anxiety, and depression. Women reported higher levels of depression and stress; job loss predicted depression and anxiety; low income predicted depression; and COVID-19-related health concerns predicted anxiety. This study highlights the unique and multiple challenges faced by minority students from disadvantaged backgrounds. Campus programs are needed to address the emotional needs of students. Longitudinal research is needed to more fully understand the impact of COVID-19 on higher-education students.

## 1. Introduction

COVID-19 is a highly infectious, novel coronavirus, SARS-CoV-2 [1]. In March 2020, WHO declared COVID-19 a global pandemic [2]. In an attempt to mitigate the spread, various measures were imposed, including social distancing, lockdowns, and quarantine (i.e., shelter-in-place). Although these reduced the spread of the virus, the resulting social and physical isolation led to various negative consequences on mental health and well-being [3]. Most studies assessing the psychological impact of COVID-19 have reported higher rates of anxiety, stress, and depression in various populations [3,4,5]. 

### 1.1. Students’ Mental Health

The prevalence of mental health issues among higher-education students is of concern as growing numbers are reporting anxiety, depression, and stress [3]. Before the pandemic, Saraswathi and colleagues (2020) found that in India the prevalence of depression, anxiety, and stress among medical students was 33.2%, 21.2%, and 20.7%, respectively, but increased to 35.5%, 33.2%, and 24.9% during COVID-19 [6]. A similar study in China found that 45% of college students reported clinical stress, anxiety, and depression; exposure to media coverage was associated with acute stress [7]. 

Psychological distress affects academic performance and can adversely impact future careers. Social isolation caused by pandemic restrictions may have caused or worsened mental health issues. To limit the transmission of COVID-19, universities and colleges suspended in-class lectures, shifting instead to online learning. Life changed drastically, as students were no longer on campus and forced to adapt to remote, online learning platforms in relative isolation. Some struggled to obtain reliable equipment and internet access [3] and others had to suspend research projects and internships, jeopardizing their studies, and delaying graduation [5,8]. These disruptions could affect career competitiveness, leaving students more worried about finances, especially those working in industries shut down in compliance with prevention protocols [8]. These changes along with social isolation have increased levels of stress and anxiety for students worldwide.

There is limited research examining the impact of COVID-19 on the mental health of students outside of the U.S. [3]. It is known that culture, ethnicity, race, and socioeconomic status each play a role in determining how individuals cope with stress and anxiety [4,5,9]. 

### 1.2. The Arab Minority in Israel

Arab Israelis constitute 21% of the Israeli population and live in all regions of the country [10]. Most are Sunni Muslims with smaller numbers of Druse and Christians. They experience socioeconomic and health inequalities at both the individual and neighborhood levels [9,11]. Compared to the Jewish majority, Arab Israelis tend to have lower education, income, and employment [12,13]. These inequalities contribute to social exclusion, oppression, low social cohesion, and racial discrimination which foster stress, anxiety, and depression [11,13]. 

The present study focuses on Arab minority students enrolled in colleges and universities in Israel. Arab-Israelis comprised 16.9% of students in higher education [14] and encounter a multitude of stressors throughout their academic careers even before the COVID-19 pandemic [13]. Some stressors are universal to all students while others are unique to the political and social context of Arabs in Israel [13]. Universal challenges faced by students include financial, academic, and social stressors as well as adapting to independent living (i.e., away from home) [15,16]. Unique stressors for Arab students in Israel include adapting to the majority culture and studying in Hebrew as well as various social, cultural, and political stressors [4,15,16]. These challenges led to higher levels of psychological distress [13].

The COVID-19 pandemic likely exacerbated stressors for Arab students in Israel [13]. The impact of the pandemic on Arab populations in Israel has been examined [13,17,18] but to our knowledge, this is the first to examine the widespread impact including stress, anxiety and depression, and academic challenges among Arab minority students in higher education in Israel. 

Thus, in this study, we set out to evaluate students’ levels of depression, anxiety, and stress. In addition, measuring the impact of the COVID-19 pandemic on the mental health of Arab students in Israel, in relation to health and academic factors, and financial factors. Identifying the impacts of the pandemic on this minority group of students can provide us with a better understanding of the mental consequences during an emergency, which in turn provides preparedness for crisis periods that higher-education institutions may face in the future. Our results could assist policymakers in developing appropriate policies that are tailored to the Arab minority population in Israel that may improve their mental health and their educational achievements.

## 2. Materials and Methods

### 2.1. Study Design and Participants

This cross-sectional study consisted of Arab undergraduate and graduate students in Israel. Prospective participants were 18+ years of age, spoke Arabic, and were registered at an Israeli institute of higher education in 2021 (e.g., university, academic college). Data were collected from January 2021 to January 2022. The resulting sample consisted of 420 students (63% B.A., 17.6% M.A., 4.3% Ph.D., 15% other) living in northern, central, and southern Israel.

### 2.2. Data Collection

Due to imposed lockdown, participants were recruited using various social media platforms such as Facebook, WhatsApp, Viber, Virtual Classrooms, and Instagram. An online questionnaire (Google Forms) was used to obtain responses. 

### 2.3. Variables and Measures

#### 2.3.1. Outcome Measures

The Depression Anxiety Stress Scale (DASS-21) is a short version of the original DASS-42 [19], developed to measure symptoms of stress, anxiety, and depression (7 items per subscale) over the past week. Each of the 21 items is scored on a Likert scale ranging from did not apply to me at all (0), to apply to me very much or most of the time (3). 

The DASS-21 has been widely used across clinical and non-clinical settings [20] and has been translated and validated in Arabic [21,22]. Internal consistency of DASS-21 responses in this study is high across subscales (0.90 < α < 0.94). 

#### 2.3.2. Independent Variable Measures

##### Socio-Demographic Characteristics

Participants reported demographic information including age, gender, region (north, center, south), relationship status (married, single, widowed, divorced/separated), Level of study (undergraduate, graduate), employment status (employed/unemployed), Income (above or below average), number of children (0 or ≥1), housing (own residence, share with others, live with family).

Economic impact of COVID-19 was measured by asking, “To what extent has the COVID-19 pandemic negatively affected your finances”? Responses were provided on a Likert scale and dichotomized between not at all to a small extent (i.e., 0–1) and moderate to a large extent (i.e., 2–3). 

Loss of job during the COVID-19 Pandemic was measured by asking, “Did you lose your job due to COVID-19”? Responses were reported as yes or no.

Academic difficulties were measured by asking, “To what extent have you had academic concerns or difficulties due to the COVID-19 pandemic”. Responses were provided on a Likert scale and dichotomized between never to a small extent (i.e., 0–1) and moderate to a large extent (2–3). 

Academic support was measured by asking, “How supported do you currently feel by your academic institution?” Responses were reported along a Likert scale ranging from not supported (0) to very supported (4), and dichotomized as high or low (i.e., above or below median score). 

Quality of online learning was measured by asking, “Based on your experience, which of the following best describes the quality of online-teaching and learning during the pandemic” ? Responses were provided on a Likert scale, and dichotomized between very low to moderate (i.e., 0–1) and high to very high (i.e., 2–3). 

Health concerns and COVID-19 was measured by asking, “To what extent do you feel concerned for your health due to COVID-19”? Responses were reported on a Likert scale and dichotomized between not at all to a small extent (i.e., 0–1) and moderate to a large extent (i.e., 2–3). 

Exposure to COVID-19: Participants indicated (yes or no) if they had been diagnosed with COVID-19, had COVID-related symptoms, had direct physical contact with an infected person, and if a relative had died due to COVID-19.

### 2.4. Statistical Analyses

Multivariate logistic regression was performed to identify predictors of depression, anxiety, and stress. All independent variables in initial analyses associated with depression, anxiety, and stress symptoms were included in the multivariate analysis, as well as variables identified in previously published research. Odds ratios (ORs) and 95% confidence interval (95% CI) were computed. The threshold for statistical significance was set a priori at *p* < 0.05. Statistical analyses were performed using SPSS v22 (SPSS Inc., Chicago, IL, USA).

### 2.5. Ethics Approval

Ethics approval for this study was obtained from Research Ethics Committee, Al-Qasemi Academy, Haifa, Israel (number 301220/21). A splash page explained the purpose and objectives of the study and emphasized that participation was voluntary, and that confidentiality would be maintained. Students consented to participate by responding yes to: “Are you willing to participate in this study voluntarily?” A list of services and resources for emotional support was provided at the end of the survey. Debriefing was made available after data collection.

## 3. Results

Most participants were female (79.8%) and in years 1 to 3 (74.3%) of their undergraduate degrees; a quarter were graduate students (25.7%). The mean age was 25.8 years (range 18–55). Most were single (59.8%) though many were married (40.2%) and had children (38.8%). Similar numbers lived with family (47%) or alone (46%); only 7% lived with other students. Many students (41.9%) were currently employed though the majority (77.1%) reported below-average income, and most (73.8%) reported a negative economic impact due to COVID-19. Half (51.4%) reported academic difficulties and only a third received academic support during the pandemic. Moreover, most (74%) reported moderate to low-quality of online learning during the pandemic. Most (76.6%) had been exposed to COVID-19 and most (53.8%) were very concerned about their health (Table 1).

**Figure 1:**
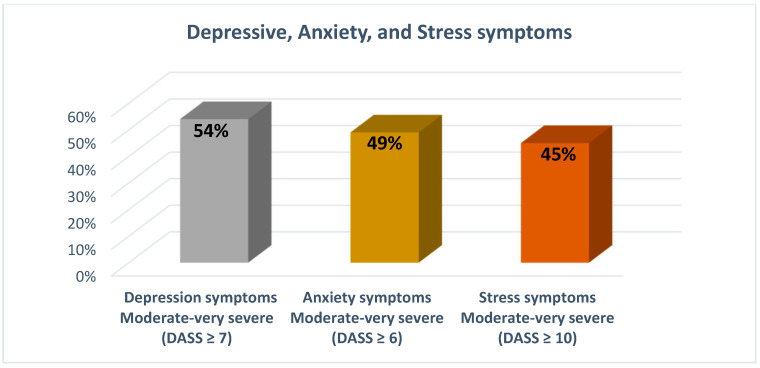
Depressive symptoms, anxiety, and stress among Arab students during COVID-19 (*n* = 420).

Figure 1 shows estimates of clinically elevated levels of depressive symptoms, anxiety, and stress. Moderate to severe symptoms of depression, anxiety and stress were reported by 49.3%, 45.2%, and 54% of Arab students, respectively.

Table 2 shows how depressive symptoms, anxiety, and stress are associated with demographic, health, and academic variables. Overall, participants reported moderate to very severe symptoms of depression, anxiety, and stress (i.e., 54%, 49.3%, and 45.2%, respectively). Women reported significantly higher symptom levels of depression and stress compared to men. Income, negative impact of COVID-19, academic difficulties, academic support, quality of online learning, and COVID-19 health concerns were found to predict depressive symptoms, anxiety, and stress. Region and job loss during the pandemic were significantly associated with symptoms of depression and anxiety, but not stress. Students from the south reported significantly higher symptoms of depression and anxiety compared to those from the center or the north of Israel. 

Table 3 presents the results of multiple logistic regression analyses with symptoms of depression, anxiety, and stress as dependent variables. Analyses indicate that low quality of online learning, academic difficulties, and negative economic impact of COVID-19 predicted stress, anxiety, and depressive symptoms. Students who reported low quality of online learning were four times more likely to report elevated symptoms of stress and depression (OR = 4.789, 95% CI = 2.80–8.26, *p* < 0.001; OR = 4.014, 95% CI = 2.3–6.9, PV < 0.001, respectively), and three times more likely to report elevated anxiety (OR = 3.2, 95% CI = 1.8–5.6, *p* < 0.001). 

Students with academic difficulties were twice as likely to report elevated symptoms of depression, anxiety, and stress (OR = 2.1, 95%CI = 1.2–3.7, *p* = 0.004; OR = 2.0, 95% CI = 1.2–3.4, *p* = 0.004; OR = 2.0, 95% CI = 1.2–3.3, *p* = 0.005, respectively). Moreover, students who reported negative economic impact of COVID-19 were at twice the risk of elevated depression, anxiety, and stress symptoms (OR = 2.2, 95% CI = 1.2–3.9, *p =* 0.005; OR = 1.9, 95% CI = 1.0–3.3, *p* = 0.030; OR = 2.1, 95% CI = 1.2–3.7, *p =* 0.005, respectively).

Female gender was a significant predictor of depressive and stress symptoms. Women were nearly twice as likely to report elevated symptoms of depression and stress compared to men (OR = 2.0, 95% CI = 1.1–3.6, *p =* 0.010; OR = 1.8, 95% CI = 1.0–3.1, *p* = 0.027, respectively. 

Students who lost employment during the pandemic were twice as likely to report elevated symptoms of depression and anxiety (OR = 1.7, 95% CI = 1.0–2.9, *p* = 0.024; OR = 1.8, 95% CI = 1.0–3.0, *p* = 0.007, respectively).

Below-average income was a significant predictor of elevated depressive symptoms. Students with low income (below average) were 2.5 times more likely to report depressive symptoms (OR = 2.5, 95% CI = 1.4–4.4, *p* = 0.002).

Students with COVID-19 health concerns were 1.8 times more likely to report elevate symptoms of anxiety (OR = 1.8, 95% CI = 1.1–2.9, *p* = 0.017).

## 4. Discussion

Arab students recruited for this study reported high levels of psychological distress during the COVID-19 pandemic. This allowed us to identify predictors of depressive symptoms, anxiety, and stress. Our findings are consistent with existing research suggesting that the COVID-19 pandemic has had a significant negative impact on the mental health of higher-education students [23,24,25].

In recent years, students’ mental health has drawn the attention of researchers in many countries [26,27]. Studies show that many students experience psychological distress [28], which significantly affects their academic success and their future interpersonal and career opportunities [29,30]. Studies conducted with minority students show that stigma, racism, and discrimination as well as economic inequalities, are associated with elevated symptoms of psychological distress and clinical conditions [31,32]. The COVID-19 pandemic posed a distinct threat to disadvantaged minorities and other vulnerable populations. Recent studies have shown that public health emergencies have a significant impact on the mental health of college students [33]. Moreover, a study investigating predictors of student mental health in nine countries during the pandemic estimated the prevalence of depression, anxiety, and stress at 40.3%, 30%, and 61.30%, respectively [25]. Rates reported by Israeli students in that international study for depression, anxiety, and stress were 42.20%, 32.70%, and 64.80%, respectively [25]. 

Various factors account for higher estimates of depression, anxiety, and stress. Predictors included: gender, income, negative economic impact of COVID-19, loss of job, academic difficulties, academic support, and poor quality of online-learning, and COVID-19 health concerns. 

Most students (73.8%) reported low to moderate quality of online learning during the pandemic. We found that elevated symptoms of depression, anxiety, and stress were significantly associated with poor quality of online learning and academic difficulties. In fact, students who reported low-quality online learning were three to four times more likely to report elevated symptoms of stress, depression, and anxiety. In addition, students who reported academic difficulties were twice as likely to report elevated symptoms of depression, anxiety, and stress. These results are consistent with recent findings with U.S. undergraduates showing that academic difficulties and challenges with online learning were significantly associated with depression, anxiety, and stress [3].

COVID-19 has redefined education, accelerating a shift to online learning [34]. And these seismic changes were undertaken with little preparation or field testing necessary to ensure accessibility. As the OECD warned, online learning may increase inequality within education systems due to poor internet access, and lack of adequate hardware or technical support [35]. 

Recent research conducted with Arab students in Israel describes Internet infrastructure issues and limited access to support services [36,37]. Just 64.8% of Arab students (vs. 87.9% of Jews) reported that the internet infrastructure in their homes was sufficient to undertake their studies (i.e., online learning and access to electronic resources). Most (83.1%) Jewish students vs. 31.3% of the Arab students connected to Zoom via their own computers, meaning that most (70%) Arab students used their cell phones for online learning. Many (40%) Arab students faced technical problems regarding remote learning compared to just 16% of Jewish students [36]. Moreover, Arab students in the south faced more challenges and difficulties during the pandemic. Most (90%) reported difficulties with online learning, 82.1% were concerned regarding the completion of their degrees, and 55.6% said that they might have to drop out. Most (65%) said they do not have reliable internet in their home [37]. 

Economic difficulties and concerns were also associated with poor mental health. Students who reported a negative economic impact of COVID-19 (e.g., loss of job) were twice as likely to experience symptoms of depression, anxiety, and stress. Students who reported below-average income were 2.5 times more likely to report elevated depressive symptoms. Similar results have been reported abroad. Kimhi (2020) reported that COVID-19 has had a global impact, affecting students’ employment and income, and possibly their ability to graduate as planned [38]. Another study regarding higher education in Israel during COVID-19 found that 74% of Arab minority students’ households were severely affected economically by the pandemic vs. 43% of the majority students’ households [36]. Further, 22% of Arab students considered dropping out compared to just 10% of Jewish students [36]. A U.S. study conducted with higher-education students found that increased depression is associated with unemployment, and lower pay [3]. 

Our findings indicate that gender is significantly associated with poor mental health. Arab women studying in Israel are nearly twice as likely to report elevated symptoms of depression and stress compared to Arab male students. Other research conducted during the pandemic has reported that women are at an increased risk of depression and anxiety [3,7,39]; and in studies conducted before the pandemic, female students consistently report higher levels of psychological distress than men [40,41,42,43,44]. COVID-19 may have more negatively affected female Arab students in Israel, who also experience gender and ethnic discrimination [45]. 

Another factor impacting students’ psychological well-being is COVID-19-related health concerns. Most Arab students (53.8%) reported feeling very to extremely concerned about COVID-19. In addition, students with COVID-19 concerns were 1.8 times more likely to report elevated symptoms of anxiety. Previous studies reveal that past pandemics (e.g., severe acute respiratory syndrome, Ebola, and Middle East respiratory syndrome), led to increased health concerns [46,47]. A systematic review of 16 studies with 11,872 college students, indicates that COVID-19 health concerns are significant factors that contribute to feelings of anxiety and depression [48]. By contrast, we found no significant associations between psychological distress and region, relationship status, number of children, level of study, and housing or academic support. 

Although these findings provide novel information about the impact of COVID-19 on the mental health of minority Arab higher-education students in Israel, several limitations should be noted. Limitations of this study include cross-sectional data collection, limiting our ability to identify causal associations. In addition, data were collected through an online survey and based on participants’ self-reports, a procedure that might result in self-report bias. Another limitation is the lack of screening for psychiatric conditions. Since participants are not physically in a face-to-face setting, it was impossible to conduct a clinical diagnosis of depression, anxiety, and stress by a physician. In addition, the use of screening measures reduces our ability to determine incidence rates of psychopathology among higher-education students. Future research should administer structured diagnostic interviews and chart reviews to better determine acute psychopathology (e.g., major depressive disorder). Nor did we have baseline, pre-pandemic information concerning the mental health of these students. Thus, we are unable to determine the extent of change over time in their mental health and well-being. For this study, we focused on one ethnic group, Arab higher-education students in Israel. Thus, we are unable to compare to other students in Israel. And though our sample included students from across the country, it is unclear to what degree these participants are representative of the population. 

## 5. Conclusions

The current study confirms that the COVID-19 pandemic has negatively affected the mental health of Arab higher-education students in Israel. This study highlights the unique challenges faced by minority students from disadvantaged backgrounds. Multiple factors, such as job loss, low income below, academic difficulties, poor quality of online learning, female gender, and COVID-19-related health concerns are associated with an increased risk of poor mental health. 

Specific programs to foster well-being and practical support for minority students are needed to promote mental health awareness, (e.g., student mental health services). It is also important to build support systems that can be mobilized in real time to address crises and the emotional needs of students on campus. To allow students to thrive academically, institutions should promote wellness and foster campus cultures that prioritize well-being as a value. To address this, academic institutions should increase awareness and address stigma to remove stigmas behind mental health issues and reduce barriers of access to mental health support facilities on campuses, while destigmatizing the need to seek mental health support. In addition, they should encourage integrated peer-to-peer programs and student-led outreach programs that can open conversations to better understand and support higher-education students’ needs. These can also help students empathize with each other, value sharing similar experiences, and raise awareness concerning available mental health resources. Moreover, we suggest that universities and colleges make advisors more accessible through technology solutions using digital mental health services via smartphone apps, through these, students can reach out and they are more likely to seek assistance. 

We identified several factors associated with high levels of mental health symptoms by students during the pandemic. These include a lack of academic support and poor quality of online learning and associated difficulties. Further effort is needed in order to make higher education more accessible to minority, vulnerable, and disadvantaged students. It is vital to encourage minority students to complete their academic studies to successfully integrate into broader society. Campuses that show dedication to student well-being can help increase academic performance, retention, and graduation rates mental health support institutions should be empowered with platforms to conduct online well-being checks. A simple questionnaire emailed to students can help the institution determine the stress levels of students while learning what difficulties they face, and what the way forward should look like in order to reduce depression, anxiety, and stress symptoms and increase the student’s success in their studies. Additionally, measuring the success of mental health programs on campuses. Further research is needed to examine the effect of COVID-19 on other student populations (i.e., other ethnic groups, age, and gender differences). Both quantitative and qualitative research is needed to fully understand the impact of the pandemic on higher education from the perspective of students, lecturers, unions, and administration. We suggest also, evaluating the effectiveness of a variety of intervention programs, e.g., online therapy, adapted to pandemic periods, or on campuses during frontal studies.

## Figures and Tables

**Table 1:** Sociodemographic, academic, and health characteristics (*n* = 420).

Characteristic	Mean (SD)/No. (%)
Age (mean [SD], range)	25.8 (7.0), 18–55
Gender	
Women	335 (79.8)
Men	85 (20.2)
Region	
North	156 (37.1)
Center	150 (35.7)
South	114 (27.1)
Relationship status	
Married	169 (40.2)
Not married (single/widowed/divorced/separated)	251 (59.8)
Number of children	
0	257 (61.2)
≥1	163 (38.8)
Level of study	
Undergraduate	312 (74.3)
Graduate	108 (25.7)
Housing	
Own residence	194 (46.2)
Share with others	30 (7.1)
Share with family members	196 (46.7)
Employment	
Employed	176 (41.9)
Unemployed	244 (58.1)
Income	
Below average	324 (77.1)
Above average	96 (22.9)
Economic impact of COVID-19 pandemic	
Not at all to a small extent	110 (26.2)
Moderate to a large extent	310 (73.8)
Loss of job during the COVID-19 pandemic	
Yes	149 (35.5)
No	271 (64.5)
Academic difficulties	
No	204 (48.6)
Yes	216 (51.4)
Academic support	
Low	302 (71.9)
High	118 (28.1)
Quality of online-learning during COVID-19	
Low-moderate	310 (73.8)
high quality	110 (26.2)
Health concerns and COVID-19	
Not at all to moderately	194 (46.2)
Very to extremely concerned	226 (53.8)
Exposure to COVID-19	
No	136 (32.4)
Yes	284 (67.6)

**Table 2:** Bivariate associations of demographic, health, and academic factors with mental health among higher-education students during COVID-19 pandemic (*n* = 420).

Variables	DASS-21 Depression Scores ≥ 7 * N = 227 (54.0%)		DASS-21 Anxiety Scores ≥ 6 * N = 207 (49.3%)		DASS-21 Stress Scores ≥ 10 * N = 190 (54.2%)	
	**N (%)**	***p*-Value**	**N (%)**	***p*-Value**	**N (%)**	***p*-Value**
Age (mean (SD))	25.2 (6.8)	0.306	26.0 (7.1)	0.627	25.7 (7.0)	0.852
Gender		0.015		0.930		0.021
Women	191 (57.0)		148 (44.2)		161 (48.1)	
Men	36 (42.4)		38 (44.7)		29 (34.1)	
Region		0.009		0.003		0.184
North	84 (53.8)		76 (48.7)		77 (49.4)	
Center	69 (46.0)		50 (33.3)		59 (39.3)	
South	74 (64.9)		60 (52.6)		54 (47.4)	
Relationship status		0.096		0.332		0.772
Married	83 (49.1)		70 (41.4)		75 (44.4)	
Not married (single, divorced, widowed, separated)	144 (57.4)		116 (46.2)		115 (45.8)	
Number of children		0.673		0.241		0.958
0	141 (54.9)		108 (42.0)		116 (45.1)	
≥1	86 (52.8)		78 (47.9)		74 (45.4)	
Level of study		0.450		0.969		0.353
Undergraduate	172 (55.1)		138 (44.2)		137 (43.9)	
Graduate	55 (50.9)		48 (44.4)		53 (49.1)	
Housing		0.075		0.671		0.443
Own residence	99 (51.0)		88 (45.4)		83 (42.8)	
Share with others	12 (40.0)		11 (36.7)		12 (40.0)	
Share with family members	116 (59.2)		87 (44.4)		95 (48.5)	
Employment		0.224		0.432		0.472
Yes	89 (50.6)		74 (42.0)		76 (43.2)	
No	138 (56.6)		112 (45.9)		114 (46.7)	
Income		<0.001		0.002		<0.001
>Average	28 (29.2)		29 (30.2)		28 (29.2)	
≤Average	199 (61.4)		157 (48.5)		162 (50.5)	
Economic impact of COVID-19		<0.001		<0.001		<0.001
No at all to a small extent	31 (28.2)		26 (23.6)		27 (24.5)	
Moderate to a large extent	196 (63.2)		160 (51.6)		163 (52.6)	
Loss of job during the COVID-19 pandemic		<0.001		<0.001		0.120
No	128 (47.2)		101 (37.3)		115 (42.4)	
Yes	99 (66.4)		85 (57.0)		75 (50.3)	
Academic difficulties		<0.001		<0.001		<0.001
None or few	76 (37.3)		57 (27.9)		61 (29.9)	
Some to many	151 (69.9)		129 (59.7)		129 (59.7)	
Academic support		<0.001		<0.001		<0.001
High	40 (33.9)		31 (26.3)		35 (29.7)	
Low	187 (61.9)		155 (51.3)		155 (51.3)	
Quality of online learning during COVID-19		<0.001		<0.001		<0.001
High	28 (35.5)		24 (21.8)		22 (20.0)	
Low (not effective)	199 (64.2)		162 (52.3)		168 (54.2)	
Health concerns and COVID-19		0.002		<0.001		0.001
Low	89 (45.9)		60 (30.9)		71 (36.6)	
High	138 (61.1)		126 (55.8)		119 (52.7)	
Exposure to COVID-19		0.465		0.871		0.913
No	77 (56.6)		61 (44.9)		61 (44.9)	
Yes	150 (52.8)		125 (44.0)		129 (45.4)	

* Moderate—very severe symptoms.

**Table 3:** Multiple logistic regression of demographic, academic and health factors, and mental health (*n* = 420).

Variables	DASS-21 Depression		DASS-21 Anxiety		DASS-21 Stress	
	OR	95% CI (*p*-Value)	OR	95% CI (*p*-Value)	OR	95% C (*p*-Value)
Gender						
Men	1 (Ref)				1	
Women	2.095	1.19–3.68 (0.010)	-	-	1.842	1.07–3.16 (0.027)
Region						
North	1		1			
Center	0.886	0.51–1.53 (0.665)	0.663	0.39–1.11 (0.119)	-	-
South	1.162	0.64–0.09 (0.616)	1.283	0.48–1.44 (0.512)	-	-
Income						
>Average	1		1		1	
≤Average	2.487	1.40–4.41 (0.002)	1.248	0.70–2.21 (0.451)	1.586	0.90–2.78 (0.107)
Economic impact of COVID-19						
Not at all to a small extent	1		1		1	
Moderate to a large extent	2.263	1.28–3.98 (0.005)	1.889	1.06–3.35 (0.030)	2.187	1.26–3.78 (0.005)
Loss of job during the COVID-19 pandemic						
No	1		1			
Yes	1.773	1.07–2.91 (0.024)	1.894	1.18–3.01 (0.007)	-	-
Academic difficulties						
None or few	1		1		1	
Some to many	2.198	1.29–3.74 (0.004)	2.067	1.25–3.41 (0.004)	2.033	1.23–3.34 (0.005)
Academic support						
High	1		1		1	
Low	1.311	0.73–2.32 (0.356)	0.608	0.65–2.06 (1.162)	1.134	0.64–1.98 (0.647)
Quality of online learning during COVID-19						
Moderate–high	1		1		1	
Low–not at all	4.789	2.80–8.26 (<0.001)	3.239	1.87–5.60 (<0.001)	4.014	2.32–6.94 (<0.001)
Health concerns and COVID-19						
Low	1		1		1	
High	1.067	0.63–1.81 (0.809)	1.805	1.10–2.93 (0.017)	1.198	0.73–1.94 (0.468)

## Data Availability

Anonymized data are available from the corresponding author on reasonable request.
